# Health-related quality of life in edentulous patients

**DOI:** 10.25122/jml-2021-0277

**Published:** 2021

**Authors:** Sibel Dikicier, Arzu Atay, Cumhur Korkmaz

**Affiliations:** 1. Department of Prosthodontics, Hamidiye Faculty of Dentistry, University of Health Sciences, Istanbul, Turkey

**Keywords:** quality of life, complete denture, systemic diseases, edentulism, DM – diabetes mellitus, OP – osteoporosis, HP – hypertension, OHQoL-UK – United Kingdom-Oral Health-Related Quality of Life, VAS – Visual Analog Scale, WHO – World Health Organization

## Abstract

This study aimed to assess Oral Health-Related Quality of Life (OHQoL) and satisfaction in patients who had complete denture treatment and were diagnosed with systemic diseases. Eighty edentulous patients using new complete dentures were separated into four groups according to their diseases [diabetes mellitus (DM)/osteoporosis (OP)/hypertension (HP)/healthy (control)]. The groups answered the Turkish version of the United Kingdom-Oral Health-Related Quality of Life (OHQoL-UK) and Visual Analog Scale (VAS) questionnaire four weeks after treatment. OHQoL-UK scores were slightly higher for females regardless of the type of disease. Compared with the control participants (57.95±5.33), the scores of the OHQoL-UK were not significantly different in OP and HP groups except for the DM group (58.7±5.37, 58.9±6.44, 45.3±5.19 respectively). DM was significantly associated with the increase of OHQoL values (p<0.05). Patients in all groups reported significantly higher “physical health” scores than other subdomains of OHQoL-UK, although positive correlations were determined among them. Presence of disease had no relationship to the VAS scores. This study shows that systemic diseases might not affect and predict patients' satisfaction with their complete dentures and OHQoL. DM is an independent risk factor for oral health. Satisfaction with the prosthesis might concern a patient's level of OHQoL.

## Introduction

The impact of health on quality of life has received increased relevance in both medicine and dentistry. The description of complete oral health is not restricted to the absence of pathology. The World Health Organization (WHO) states that “health is a state of complete physical, mental and social well-being and not merely the absence of disease or infirmity” [1]. This idea has led to a multidisciplinary approach to the concept of health.

Quality of life is prominently affected by oral health in the majority of people. A variety of patient-centered outcome instruments connected with oral health-related quality of life (OHQoL) have been developed to evaluate the influence of oral health problems on nutrition, pain, and other fields such as social functioning and wellness [2]. The concept of OHQoL is based on three assumptions of interaction with each other: (a) results of oral and dental health assessments, (b) conditions affecting oral and dental health also affect general health, and (c) systemic diseases and general health affects the OHQoL. The first assumption proposed is that the oral health of any individual should be at an acceptable level. The second is based on the assumption that oral and dental health is a component of general health and contributes to OHQoL. In the third assumption, it is emphasized that oral and dental health affect general health, and like many systemic diseases cause complications in the oral cavity. Adverse oral conditions negatively affect the OHQoL and general health-related quality of life [3, 4].

Dentists want to know about patients’ satisfaction with denture and usage [5]. Multiple factors, such as mastication, taste, pain, phonation and aesthetics, could simultaneously affect different aspects of OHQoL and satisfaction with the prosthesis [6]. In addition, many systemic diseases and related long-term medications that are commonly encountered in edentulous patients with conventional complete dentures may be linked to oral health alterations [7]. Currently, developed technology has facilitated the successful treatment of these diseases. However, there are still many problems, including dental health, among these patients [8].

The literature emphasizes that traditional assessment methods of health data are not fully adequate to measure the effects of treatment on health status. Therefore, patients’ opinions related to OHQoL are increasingly important in terms of treatment when examining OHQoL assessment methods in prosthetic treatments [9].

OHQoL-UK, recently developed in the United Kingdom, is based on the WHO model of “structure-function-ability-participation” which incorporates negative and positive health influences [10]. These measures differ in their primary theoretical frameworks, dimensions, and domains and have been confirmed in the partial setting. The OHQoL-UK has been used to study the correlation between OHRQoL and prosthodontic treatment.

VAS (Visual Analog Scale) is a useful instrument for surveying the perception of QoL in dental health conditions. It is used for research purposes but also for patients seeking dental treatment to assess pre- or post-operative feelings. The assessment of a patient’s level of satisfaction on the VAS helps clinicians think according to the patient’s mindset [11].

Although the literature contains many studies exploring the effects of prosthetic treatment and/or edentulism on OHQoL as assessed by questionnaire techniques [8, 12, 13], many studies related to systemic diseases are primarily focused only on the effects of diabetes mellitus [14–16]. In the present study, we aimed to determine the relationships between edentulous patients’ satisfaction with new complete dentures and OHQoL in patients diagnosed with common systemic diseases [diabetes mellitus, hypertension, and osteoporosis], using current and reliable dental and psychological questionnaires. We initially hypothesized that systemic disease patients with complete dentures would have lower OHQoL scores than the control group patients.

## Material and Methods

### Participants’ characteristics and study design

Study participants were edentulous patients needing complete denture treatment, consulting the dental clinic during a 6-months period. The sample included eighty participants who were treated with new complete dentures, sixty in each of three patient groups, defined by their diagnosis with one of the following systemic diseases [diabetes mellitus (DM), hypertension (HP), osteoporosis (OP)], and twenty in healthy (control) participants. A standard systemic examination was carried out in all participants, the presence/absence of medical treatments was also registered. Individuals were included in disease groups based on self-reports about their medical conditions. Differences in medical treatments (diet, tablets, injections) were not included in the study as a variable.

Patients were selected by the same clinician according to the following criteria. The inclusion criteria were: (a) aged 40 years and older and edentulous, (b) at least one month experience with wearing new complete dentures, (c) an adequate level of cognitive ability and literacy to complete the questionnaire, (d) for working groups, diagnosed with any of the diseases of DM, OP or HP (e) volunteering to participate in the study. Exclusion criteria were: (a) not being treated with new complete dentures for at least the last one month for working groups, (b) diagnosed with two or more of the diseases, (b) insufficient cognitive ability and an inadequate level of literacy to complete the questionnaire, (c) not volunteering to participate in the study.

Each patient was evaluated completely to record the condition of a prosthesis. Only patients clinically in need of a complete denture were included. These patients were divided into four groups, according to the diagnosed systemic diseases, as follows: (a) group DM included patients diagnosed with diabetes mellitus, (b) group OP included patients diagnosed with osteoporosis, (c) group HP included patients diagnosed with hypertension and (d) control group included healthy patients. Histories of systemic disease were determined through the patient’s own statements and were evaluated for the observation of the oral symptoms profile through a self-reporting questionnaire. Patients with incomplete medical examination, missing information or lacked a research consent form were not included in the study.

### Measurements

All participants were called four weeks after receiving new complete dentures. Data were collected using a questionnaire consisting of two parts. The first part contained the Turkish version of the OHQoL-UK [17], and the second part comprised the VAS scale. The Turkish adaptation of the OHQoL-UK scale was achieved by Mumcu *et al.* [17]. McGrath and Bedi originally developed OHQoL-UK by talking to patients [18]. It consists of 16 items with a Likert response format ranging from very bad effect (score 1) to very good effect (score 5) in three subdomains: physical health (7 items), psychological health (5 items), and social relationships (4 items). Participants were asked items like: “How would you rate the impact of this effect on your overall quality of life? ’very bad, bad, no, good, or very good effect?” [19]. The total score ranges between 80 (best possible effect on OHQoL) and 16 (worst possible effect on OHQoL). In addition, summing up responses to items in each domain can produce subdomain scores. The OHQoL-UK measures relative positive assessments of individuals’ perception of oral health and the negative impacts [19, 20]. It is a current safe, and sensitive questionnaire to assess OHQoL [10].

A single-measure horizontal VAS scale questionnaire, from 0 to 10 cm, structured with seven questions, was used. It assessed bother or satisfaction regarding patient QoL specific to each of the 7 questions. The ends were marked “not good” and “very good”. The questions were as follows: “Is your prosthesis comfortable?” “How is your speech with your prosthesis?” “How is the retention of your prosthesis?” “How is the aesthetic of your prosthesis?”, “Is your prosthesis easy to clean?” “How is your chewing with your prosthesis?”, “How is your prosthesis in general?”. Each question is scored (from 0 to 10), and scores for the scales range from 0 to 70. Patients were asked to mark their level of satisfaction on the line. The span was measured and recorded.

Patients were asked to complete these questionnaires in the waiting hall. If they were unable to understand, they were assisted by a dental assistant trained for that study available for explanations during questionnaire administration.

### Data analysis

The answer rate to the questionnaire was calculated, and frequency tables were prepared to investigate the prevalence of effects and their action on OHQoL. Variations in OHQoL-UK scores and their subdivisions (physical, psychological, and social) related to social factors and systemic diseases were examined. There was no incomplete data for any questions on the OHQoL-UK and VAS. These confirm the good compatibility between the instruments and participants.

The data were analyzed using SPSS (Statistical Package for the Social Sciences, version 11.0, SPSS Inc., Chicago, IL). Characteristic data on frequencies, percentages, means, and standard deviations were achieved according to the socio-demographic and clinical specifications of the participants. The associations between the variables were analyzed using the Pearson correlation test, and ANOVA was used to compare different groups. Kruskal–Wallis analysis and Mann–Whitney U test with Bonferroni correction were used in cases where the data were not normally distributed. For all statistical analyses, the significance level was set at p≤0.05.

## Results

The demographic characteristics and clinical conditions of the patients’ group are shown in Table 1. There were significant differences between the groups according to age and gender variables beyond the type of dentures (p<0.05). The mean OHQoL-UK scores are reported in Table 2 for each group according to their new complete denture experience. There were no statistical differences between the systemic disease groups and the control group in HP and OP for overall OHQoL-UK scores. Patients with DM showed significantly lower values of QOL compared to healthy patients (p<0.05).

**Table 1.** Participants' characteristics and comparisons between the groups (n=80).

	**DM**	**OP**	**HP**	**Control**	p *
**Characteristics**					
Age(years) mean(SD)	64.2(7.9)	66.95(8.92)	67.6(6.48)	61.2(8.31)	0.038 ^a^
**Gender (n,%)**					
Female	2 (10%)	16 (80%)	6 (30%)	6 (30%)	<0.000 1 ^a^
Male	18 (90%)	4 (20%)	14 (70%)	14 (70%)	
**Type of dentures (n,%)**					
Uni-maxillary	5 (25%)	14 (20%)	2 (10%)	4 (20%)	0.67 ^a^
Bi-maxillary	15 (75%)	16 (80%)	18 (90%)	16 (80%)	

DM – group diabetes mellitus, OP – group osteoporosis, HP – group hypertension, SD – standard deviation; * – p<0.05; ^a^ – chi-square test.

**Table 2.** Comparison of OHQoL-UK and subdomains scores between groups after prosthetic rehabilitation.

**Domains**	**DM** **n=20** **Mean(SD)**	**OP** **n=20** **Mean(SD)**	**HP** **n=20** **Mean(SD)**	**Control** **n=20** **Mean(SD)**	p *	**Multiple** **comparisons**
**Overall OHQoL-UK**	45.3 (5.19)	58.7 (5.37)	58.95 (6.44)	57.95 (5.33)	0.78 ^a^	<0.05 ^b^
**Physical**	26.8 (3.04)	26.9 (2.77)	26.8 (2.65)	27.45 (3.14)	0.87 ^a^	>0.05 ^b^
**Psychological**	16.05 (1.93)	17.25 (2.31)	17.5 (2.42)	16.2 (1.28)	0.07 ^c^	>0.05 ^d^
**Social**	14.05 (1.67)	14.4 (1.76)	14.15 (1.6)	14.3 (1.92)	0.07 ^c^	>0.05 ^d^

Higher scores indicate greater QoL; DM – group diabetes mellitus, OP – group osteoporosis, HP – group hypertension, SD – standard deviation * – p<0.05; ^a^ – One-way ANOVA; ^b^ – Bonferroni; ^c^ – Kruskal Wallis analysis d Bonferroni corrections Mann Whitney U.

As shown in Table 2, the following post hoc comparisons using the Bonferroni test showed that patient groups did not have significant differences for subdomain scores. Physical subdomain had significantly higher scores in all patient groups than other OHQoL-UK subdomains (p<0.001). After new complete denture treatment, the total mean scores for both the OHQoL-UK and subdomains were higher in females regardless of a systemic disease but not statistically significant when compared with males (Table 3).

**Table 3.** Gender characteristics associated with QoL in patients regardless of systemic diseases.

	**Gender**		p *
	**Female**	**Male**	
	**Mean(SD)**	**Mean(SD)**	
**OHQoL-UK**	60.53 (5.95)	56.84 (4.82)	>0.05
**Physical**	28.10 (2.94)	26.32 (2.62)	>0.05
**Psychological**	17.30 (2.38)	16.42 (1.85)	>0.05
**Social**	14.63 (1.65)	13.98 (1.72)	>0.05
**VAS**	49.16 (5.82)	50.13 (4.54)	>0.05

* – p<0.05.

**Table 4.** Comparative evaluation of VAS, OHQoL-UK and subdomains’ scores, between groups related with gender.

**Gender**	**OHQoL-UK** **Mean(SD)**					**VAS** **Mean(SD)**				
	**DM**	**OP**	**HP**	**control**	p *	**DM**	**OP**	**HP**	**control**	p *
**Female**	50.01 (2.82)	59.06 (5.53)	64 (8.64)	61.66 (2.42)	0.94 ^a^	53.85 (1.62)	47.64 (6.24)	50.13 (2.86)	50.65 (7.10)	0.10 ^a^
**Male**	52.29 (5.24)	57.25 (5.12)	57.56 (5.79)	56.35 (5.48)	0.34 ^a^	53.33 (5.03)	53.1 (1.85)	50.11 (5.00)	51.10 (4.58)	0.88 ^a^

* – p<0.05; ^a^ – One-way ANOVA test.

VAS scores for OP were 48.74.2±6.03 and lower than DM, HP, and control (49.23±4.38, 50.12±4.4, and 50.97±5.26, respectively). However, VAS scores for systemic disease groups that used new complete dentures did not differ significantly (p=0.52) from the control group. According to VAS, “chewing efficiency” and “retention” items related to issues with the complete dentures showed lower scores than other items. There were no significant differences between female and male patients in VAS scores. In addition, for all patient groups, gender did not significantly influence the OHQoL-UK, and VAS mean scores (Table 4).

A strong positive correlation was found between OHQoL-UK total scores and subdomains: physical (p=0.001, r=0.76), psychological (p=0.001, r=0.62), and social (p=0.001, r=0.68). Within OHQoL-UK subdomains, a significant positive correlation was found between physical and psychological, psychological and social, physical and social scores (p=0.001; r=0.35, 0.44, and –0.45, respectively). The best OHQoL (maximum scores of OHQoL-UK) were associated with the highest levels of total satisfaction and aesthetics. On the other hand, no significant correlation was found between OHQoL-UK and VAS scores (p>0.05).

## Discussion

QoL may become increasingly worse in total edentulism, leading to a complex situation for general health in this population. Systemic diseases may contribute to oral health status, especially among the edentulous patients, with mastication and nutritional problems, and with highly negative effects on their QoL [20]. The oral cavity provides vital phonation, respiratory, and digestive functions and is a window into the health of the human body. Because of its anatomical position and hard and soft tissues, the oral relevance of chronic conditions are well known. In addition, some systemic diseases may first be diagnosed with oral findings [21]. DM, HP, and OP are the most frequent systemic diseases, selected in the current study, that impair oral health and have been associated with some oral symptoms including decreased quality/quantity of saliva, altered microvascularization, reduced bone mineralization, loss of alveolar supportive tissue, neuropathy etc [22]. There is evidence that many edentulous patients diagnosed with systemic diseases may indicate a reduced OHQoL [23]. In contrast, an interesting finding in our study was that OP and HP had no relationship to the current results of the OHQoL-UK questionnaire. It was revealed that patients with selected systemic diseases had similar levels of OHQoL. Also, control subjects with no systemic disease had similar QoL with these systemic disease groups. This result may be explained by the fact that that prosthetic rehabilitations restore optimum oral status and therefore increase the effects of systemic disease on the oral cavity, satisfaction, and OHQoL.Complete denture treatments were performed by the same prosthodontist and dental technician in all disease groups in the current study. The absence of a significant difference in OP and HP groups may suggest that oral health is not affected by symptoms in individuals with these diseases. The perspective in this study was to evaluate the impact of systemic disease during prosthetic use. Precisely, future research should consider the impact of all aspects of patients’ satisfactions on QoL among edentulous patients. In one way, the results in this study suggested that a new complete denture reformed patients’ OHQoL. However, patients with new maxillary or complete mandibular dentures scored the highest on physical appearance.

It is estimated that 17% of the Turkish population suffers from hypertension and 13.7% of the Turkish population over 15 years old is diabetic [24]. Also, the incidence of osteoporosis is 25% in their 50’s [25]. There is little data on the association between complete denture treatment and OHRQoL in individuals with systemic diseases, including HP and OP. Moreover, most studies have included DM, associated with periodontal conditions, and its effects on the OHRQoL. DM is associated with various oral mucosal changes, including xerostomia, denture stomatitis, possible candida colonization, and dry mouth [26, 27]. Based on these data, the authors identified that lower QoL values in DM denture wearers might be caused by impaired denture stability and irritation due to diabetes-induced bad oral condition. This was confirmed by the fact that in the current study, the OHRQoL-UK of diabetics was lower among healthy patients upon complete denture treatment, confirming the findings of a similar previous study [28].

The mean OHQoL scores for HP and OP patients’ were also not significantly different after prosthetic treatment, showing personal-reported acceptable oral health. Although patients with prostheses had high QoL, their outlook, social and functional satisfaction increased similar to their OHQoL scores after receiving prosthetic treatment. Furthermore, Veyrune *et al.* [29] reported the agreement between the dentist and the patient, the period of prosthetic work needed, and the form of new occlusal arrangement as efficient factors on after-treatment QOL scores. The reason for our results may be that participants know how to use and handle their complete dentures. These results were expected, given that dental prosthetic management requires a complete oral rehabilitation process, including systematic follow-up, patient training, special care, and alignment or renewal of the rehabilitation if necessary, all of which were applied in this patient group.

Oral health is a main element in protecting and supporting general health and well QoL. Inglehart and Bagramian [4] reported that oral health condition was nearly related to QoL, and that problems in oral health could severely reduce a patient’s QoL. However, Gregory *et al.* [30] stated that QoL could change according to patient senses. Patients’ perceptions of new complete dentures are the main components in evaluating clinical success. However, assessment of patient’s results such as health-related QoL in clinical experience may supply important data for planning proper prosthetic rehabilitation. In the present study, clinical success was processed from the patient’s perspective, and we used each patient’s first evaluation after new complete dentures.

Current results indicate that there were no significant differences between the OHQoL-UK scores of males and females. Additionally, it was found that OHQoL-UK scores of females were lower in a previous study, not in agreement with our study [6]. QoL of females showed to be more sensitive to corruption by oral status.

The VAS questionnaire used in this study consists of specific questions, and it is preferred in the assessment of complete-denture wearers. The study also identified that there was no direct relation between OHQoL-UK scores and VAS scores (total scores and satisfaction with each dimension). Additionally, some items, such as “chewing performance” and “retention problems”, were significantly decreased. These results, focused on patient-reported conclusions in VAS, may consider that the OHQoL was slightly altered in complete denture patients with systemic diseases. There are widely used modalities to measure health-related QoL for patient groups that allow a comparison between patients or with the social community [31]. The OHQoL-UK achieves to evaluate oral health both positively and negatively, while the other scales measure only negative effects [12]. This may be an insufficient measurement to evaluate the current conditions of health and wellness. Investigating positive interactions of oral health may be more appropriate in social research, as we did in this study. As a result, many patients have comparatively healthy clinical oral health levels and enjoy favorable experiences. On the other hand, the various differences indicate that patients’ prospects are not precisely determined, likely because self characteristics about OHRQoL were not taken into consideration when treatment alternatives were planned. As an option, a qualitative study focused on patients’ experiences could help define different types of patients and determine proper treatments.

Patients’ perceptions of treatment with removable prostheses are key elements in evaluating quality of life care.Taking into account patient outcomes such as health-related QoL in clinical practice may provide information for planning and evaluating extensive denture rehabilitation. Although the model created in the study included systemic diseases common in the population, the number of participants was one of the limitations of the current study.

## Conclusion

Findings from the present study are effective for complete denture rehabilitation on OHRQoL in edentulism with systemic diseases. Systemic diseases such as DM, OP, and HP share similar risk factors, and it can be emphasized that they are increasingly changing OHRQoL. Therefore, further investigations are necessary, including patients with different systemic conditions to highlight the significance of edentulism and other oral pathologies in OHRQoL.

Within the limitations of the present study, it can be concluded that the general systemic diseases and/or their corresponding medication might not be associated with QoL alterations in complete denture patients. Moreover, diabetic patients did not show acceptable oral health status, and to some extent, oral problems affected OHRQoL. The complications of diseases or side effects on the oral cavity may require clinicians to take some precautions concerning the treatment protocol.

## Acknowledgements

### Conflict of Interest

The authors declare that there is no conflict of interest.

### Ethical approval

Ethical clearance was obtained from the institutional ethical committee (46418926/18/30/06.02.2018).

### Consent to participate

Each participant signed a consent form, and the information form was also recorded, including dental and medical histories and socio-demographic status of the patient before being recruited into the study.

### Authorship

SD, AA designed the study and analyzed the data, AA supervised the study, SD, CK conducted the literature review and collected and processed data. SD and AA wrote the manuscript. All authors provided critical review to the study.
